# NIH Stroke Scale and age predict early post-stroke cognitive impairment

**DOI:** 10.3389/fstro.2026.1762758

**Published:** 2026-04-28

**Authors:** Faddi Saleh Velez, Cameron D. Owens, Jennifer Hotson, Andrea Loggini, Ana Luyza Oliveira Santos, Demian Rudyk, Daniela Mercado Pena, Melba Zuniga-Gutierrez, Maria Cedeno-Bruzual, Laura Boada Robayo, Cheyenne Gutierrez, Kate Singleton, Zyanna Stuart, Evgeny Sidorov, Andriy Yabluchanskiy, Camila Bonin Pinto

**Affiliations:** 1 Brain Stimulation and Neurorehabilitation Laboratory, Department of Neurology and Department of Neurosurgery, University of Oklahoma Health Sciences, Oklahoma City, OK, United States; 2 Vascular Cognitive Impairment, Neurodegeneration and Healthy Brain Aging Program, Department of Neurosurgery, University of Oklahoma Health Sciences, Oklahoma City, OK, United States; 3 Department of Neurology, University of Michigan, Ann Arbor, MI, United States; 4 Brain and Spine Institute, Southern Illinois Healthcare, Carbondale, IL, United States; 5 Department of Neurology, University of Oklahoma Health Sciences, Oklahoma City, OK, United States

**Keywords:** age, discharge disposition, length of stay, multivariable modeling, NIH Stroke Scale, post-stroke cognitive impairment

## Abstract

**Introduction:**

Acute post-stroke cognitive deficits, which may precede formal post-stroke cognitive impairment (PSCI), lacks evidence-based interventions or guidelines. Up to one-third of PSCI patients progress to dementia within 5 years. Deficits often emerge within 2 weeks, underscoring the need for early recognition of risk factors to guide prevention and management. However, predictive value of demographic, clinical, and stroke-specific factors remains inconsistent. We evaluated cognitive outcomes (Montreal Cognitive Assessment) at discharge and identified predictors of low function.

**Methods:**

In this retrospective study at the University of Oklahoma Medical Center, we reviewed 964 stroke patients, with 168 meeting inclusion criteria. Early PSCI was defined as Montreal Cognitive Assessment < 26 at hospital discharge post-stroke. We calculated prevalence, performed univariable logistic regression, and developed multivariable logistic regression models with and without interaction terms.

**Results:**

Patients with Montreal Cognitive Assessment < 26 were older, had longer hospital stays, higher NIH Stroke Scale, and more often discharged to non-home settings. Prevalence of early PSCI was higher with age, longer stays, non-white race, higher NIH Stroke Scale, and non-home discharge. Univariable analyses revealed length of stay, discharge disposition, NIH Stroke Scale, and race as strongest associations. Final multivariable model (NIH Stroke Scale, age, length of stay, discharge disposition) demonstrated NIH Stroke Scale and age as significant predictors, with good discrimination [Receiver Operating Characteristic-Area Under the Curve (ROC-AUC) = 0.79]. Models including interaction terms performed similarly (ROC-AUC = 0.78).

**Conclusion:**

Consistent with prior work, NIH Stroke Scale and age emerged as the most robust predictors of early PSCI. Length of stay, discharge disposition, and race were associated with early PSCI in univariable analyses and warrant further evaluation in larger prospective studies.

## 1 Introduction

Globally, advancements in acute stroke care have improved outcomes through better risk factor management and the availability of acute interventions. However, stroke remains a leading cause of morbidity worldwide, with post-stroke cognitive impairment (PSCI) affecting nearly 70% of survivors (Rost et al., 2022). PSCI, defined as cognitive impairment occurring within 6 months post-stroke (Rost et al., 2022), with cognitive deficits often manifesting within the first 2 weeks (Jaillard et al., 2009). Notably, many of the affected patients never fully regain their pre-stroke cognitive function with up to one-third progressing to dementia within 5 years (El Husseini et al., 2023).

Unlike motor recovery and secondary stroke prevention, PSCI management lacks specific interventions and guidelines (El Husseini et al., 2023), largely due to its complex pathophysiology and associated comorbidities. Moreover, no effective symptomatic treatment exists. Randomized controlled trials have failed to demonstrate meaningful cognitive benefits from pharmacological therapies (e.g., memantine, acetylcholinesterase inhibitors) (Li et al., 2023; Kim et al., 2020) or cognitive rehabilitation (O'Donoghue et al., 2022). Consequently, PSCI patients remain vulnerable to long-term cognitive decline, underscoring the urgent need for targeted therapeutic strategies.

Identifying risk factors for PSCI is critical for optimizing management and long-term care strategies. Both modifiable and non-modifiable factors influence PSCI risk and progression (Wei et al., 2024; Filler et al., 2024). Among non-modifiable factors, advanced age and pre-stroke cognitive status (Filler et al., 2024) are consistently associated with cognitive decline. Modifiable factors, including hypertension, smoking, metabolic syndrome, and diabetes, not only contribute to increased risk of stroke (O'Donnell et al., 2016) but also heighten PSCI risk (Filler et al., 2024). Furthermore, underlying vascular pathology, such as white matter hyperintensities, lacunes, and cerebral microbleeds, can further exacerbate cognitive impairment (Filler et al., 2024). However, the independent contributions of these risk factors remain a subject of ongoing debate (Filler et al., 2024).

Among potential predictors, stroke severity, measured by the National Institutes of Health Stroke Scale (NIHSS), is one of the strongest determinants of post-stroke outcomes, including cognitive function (Filler et al., 2024; Sharma et al., 2020; Adams et al., 1999). Higher NIHSS scores at admission are consistently linked to poorer functional and cognitive recovery, highlighting the impact of initial stroke severity on long-term cognitive prognosis. However, despite NIHSS well-established predictive value, its interactions with demographic and clinical risk factors remain unclear and require further elucidation (Wei et al., 2024; Aam et al., 2021). A deeper understanding of these relationships is essential for refining screening strategies and optimizing post-stroke cognitive rehabilitation efforts.

Due to cognitive deficits often manifesting early in the PSCI continuum, we aimed to evaluate cognitive outcomes at discharge [early PSIC (EPSCI)] and identify key factors associated with low cognitive function by examining demographic, clinical, and stroke-specific characteristics. By modeling these variables, we sought to refine PSCI risk stratification, improve early recognition, and inform both preventative and symptomatic treatment strategies. Specifically, we investigated the relationship between stroke severity, demographic variables, and cognitive function at discharge, with a particular focus on the predictive value of NIHSS scores. We aimed to (1) examine the prevalence of EPSCI dependent on modifiable and non-modifiable risk factors, (2) determine modifiable and non-modifiable risk factors that independently predict EPSCI after adjusting for potential confounders; and (3) determine whether key interactions in multivariable modeling influence the odds of EPSCI.

## 2 Methods

### 2.1 Standard protocol approvals, registrations, and patient consents

This retrospective study was approved by the University of Oklahoma Health Sciences Center Institutional Review Board (IRB#16195) and consent was waived. The data for this study were extracted from the Electronic Health Records (EHR) database, which included comprehensive medical data and imaging assessments. Inclusion criteria for this study were patients who had suffered a stroke of any subtype (i.e., ischemic, hemorrhagic) and had complete data, which included Montreal Cognitive Assessment (MoCA) scores at discharge, NIHSS, length of hospital stay, anatomical location of lesion and vessel territory. Patients were excluded if any of the above data were incomplete. We analyzed the medical records of 964 stroke patients admitted to the University of Oklahoma Medical Center between June 2022 and July 2023 and 168 met inclusion criteria and were included in data analysis. Patients not included in the final analysis were excluded because a MoCA was not completed at discharge.

### 2.2 Montreal Cognitive Assessment (MoCA)

The MoCA, a widely used and validated screening tool for cognitive impairment and dementia, evaluates eight cognitive domains: executive function, visual-spatial ability, memory, attention, concentration, working memory, language, and time/spatial orientation. This study employed the MoCA to assess cognitive performance at discharge following acute stroke. To ensure that the evaluation was tailored to the needs of patients with motor impairments, we employed a previously described MoCA adaptation (Dawes et al., 2019). With this adaptation, the visuospatial domain was excluded and reduced the maximum possible score from 30 to 25, with scores of 22 or higher considered within normal range. When MoCA for the blind (22-point) was administered, ≥19 was considered within normal range.

All MoCA scores not on the 30-point scale [i.e., MoCA for the blind (22-point scale), visuospatial domain removed (25-point scale)] were converted to the 30-point scale using the formula: (Raw score^*^30)/*total*. The denominator is the total raw points available on 22- or 25-point scale. These conversions are consistent with the previously validated conversion chart (Melikyan et al., 2021). In the current study, any score less than 26 (on 30-point scale) is considered EPSCI (Nasreddine et al., 2005; Owens et al., 2024).

### 2.3 Calculation of MoCA < 26 prevalence proportion (%)

The prevalence of EPSCI was determined by calculating the proportion of patients within each category who exhibited cognitive impairment. To compute the prevalence, patients were first grouped into categories based on age, stroke severity (NIHSS score), and length of hospital stay. The same methodology was applied to categorical variables such as race, discharge status, stroke location, vessel involvement, and laterality.

For each category, the prevalence proportion percentage was calculated using the formula:


$$\begin{array}{l} PSCI\;Prevalence\;Proportion\;\%=(Number\;of\;patients\;with\;MoCA\\ \;<26\;in\;category)/(Total\;number\;of\;patients\;in\;that\;category) \end{array}$$

### 2.4 Descriptive statistics and group comparisons

Participant demographic (e.g., age, ethnicity) and clinical characteristics (e.g., lesion localization, vessel territory) are presented as means and standard deviations or percentages for continuous and categorical variables. Group comparisons were conducted between patients with MoCA < 26 and MoCA ≥26 by Chi-Square test of independence or Fisher's exact for categorical variables, and unequal variance *t*-test (Welch's *t*-test) for continuous variables. Normality and homogeneity of variance was determined by Shapiro–Wilk test and Levene's test, respectively. Statistical significance was determined using a *P*-value threshold of less than 0.05 for all tests. Given the limited sample size of patients with a prior history of dementia, statistical comparisons were not performed. Instead, the data are presented descriptively to aid interpretation across the study sample. Statistical comparisons were not performed in the dementia group (*n* = 12) because the small sample size yields unstable estimates, reduces power, and increases the risk of type II (false negative) errors, making true differences or associations difficult to detect. Statistical comparisons were only performed in patients with no history of cognitive impairment during retrospective review. All analyses were performed using Python version 3.8 (Python Software Foundation) with relevant packages, including pandas, numpy, scipy, and statsmodels.

### 2.5 Univariable logistic regression analysis

A univariable logistic regression analysis was conducted to evaluate the association between each predictor variable and the likelihood of cognitive impairment, defined as MoCA < 26. The analysis was performed independently for each variable to estimate the unadjusted odds ratio (OR), corresponding 95% confidence intervals (CI), and *P*-values. Ethnicity was excluded from this analysis due to a substantial imbalance in sample size (*n* = 4 Hispanic or Latino, *n* = 164 non-Hispanic or Latino). This decision was made to avoid inflated odds ratios and potentially spurious associations arising from the very small subgroup size. Categorical variables were encoded into dummy variables, with one category serving as the reference for comparison. Continuous variables were analyzed on their original scales, assuming linearity with the log-odds of the outcome. Odds ratios and 95% confidence intervals were calculated for each variable using logistic regression. A *P*-value < 0.05 was considered statistically significant. All statistical analyses were performed using Python's scikit-learn library.

### 2.6 Multivariable logistic regression

To develop an interpretable and statistically robust model of EPSCI, we combined L1-regularized logistic regression (Lasso) with traditional statistical inference. Lasso regression, implemented with cross-validated Receiver Operating Characteristic-Area Under the Curve (ROC-AUC) scoring, was first used to identify influential variables by shrinking uninformative coefficients to zero. Separately, a full logistic regression model was fitted using the statsmodels package to identify statistically significant predictors based on *P*-values and 95% confidence intervals.

The final model incorporated the union of features selected by both approaches and was re-fit using statsmodels.Logit. Potential interaction terms between key predictors were explored in separate model iterations. Models with and without interactions were compared based on AUC-ROC and interpretability. This hybrid approach integrates variable selection with inferential modeling, supporting both clinical insight and methodological transparency.

Model performance was evaluated using the Area Under the Receiver Operating Characteristic Curve (AUC-ROC) to assess discrimination. For each predictor in the final model, coefficients, adjusted odds ratios (OR), and 95% confidence intervals (CI) were reported.

All analyses were conducted in Python version 3.8. Data manipulation was performed using the pandas library; Lasso regression and model evaluation were implemented using scikit-learn; traditional logistic regression modeling was conducted using statsmodels; mathematical computations were supported by numpy; and data visualization was performed using matplotlib and seaborn.

## 3 Results

### 3.1 Patients with early post-stroke cognitive impairment exhibit higher NIHSS, older age, longer hospital stay and non-home discharge disposition

**Figure 1:**
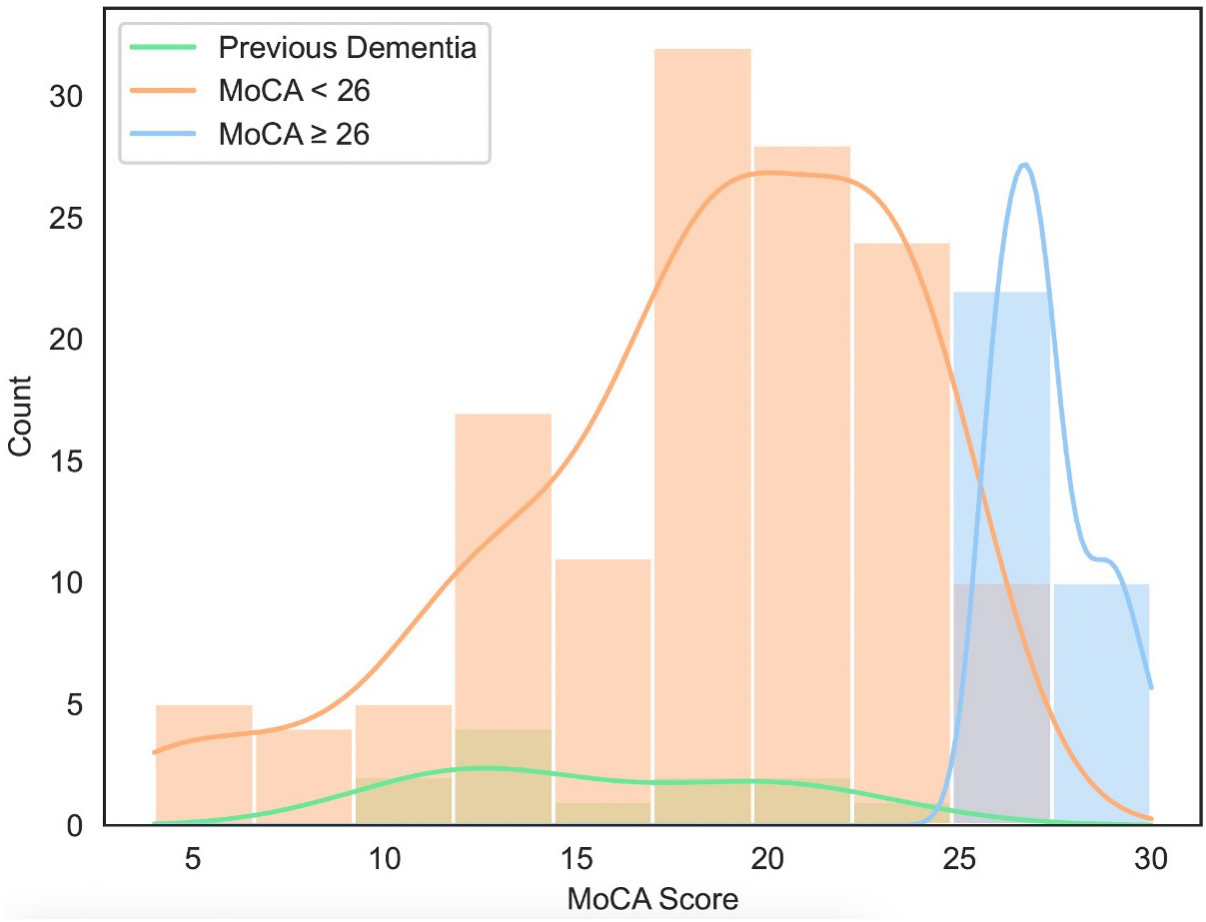
Distribution of MoCA scores in post-acute stroke patients. Patients with Montreal Cognitive Assessment (MoCA) greater than or equal to 26 is represented by the blue bar graph (*n* = 32), less than 26 by the orange bar graph (*n* = 136) and patients who had history of dementia prior to stroke have their MoCA scores represented by the green bar graph (*n* = 12). Kernel density estimation line traces are represented by each groups color designation.

To determine characteristic differences between post-stroke patients with cognitive deficits and those without, we retrospectively identified demographic (Table 1) and clinical stroke information (Table 2) from electronic medical records from OUHSC between June 2022 and June 2023. Distribution between groups with MoCA ≥26, MoCA < 26, and MoCA scores from patients with previous history of dementia are shown in Figure 1. The mean age of patients with MoCA scores ≥26 (*n* = 32) was 59.03 ± 13.7 years, while the mean age of patients with MoCA scores < 26 was 65.54 ± 13.2 years (*n* = 136; *P* = 0.019). Patients with MoCA scores ≥26 had a mean length of stay of 3.16 ± 3.3 days, whereas those with MoCA scores < 26 had a longer mean length of stay of 5.74 ± 6.6 days (*P* = 0.002). The mean NIHSS score was lower in patients with MoCA scores ≥26 (2.84 ± 5.3) compared to those with MoCA scores < 26 (6.30 ± 5.3, *P* < 0.001). Patients with MoCA scores < 26 were more likely to be discharged to a non-home setting compared to those with MoCA scores ≥26, who were more frequently discharged home (*P* < 0.001) Other categorical variables, including sex, race, ethnicity, stroke location, vessel category, side category, stroke subtype, and thrombolytics category, did not significantly differ between MoCA groups (< 26 and ≥26).

**Table 1:** Patient demographics and cognitive outcomes.

Variable	MoCA ≥26	MoCA < 26	*P*-value	Previous dementia
Age	59 (13.7)	65.5 (13.2)	0.019^*^	72.2 (13.1)
MoCA	27.3 (1.3)	18.3 (5.2)	<0.001^*^	15.8 (4.4)
Length of stay	3.2 (3.3)	5.7 (6.6)	0.002^*^	5.3 (4.5)
NIHSS score	2.8 (5.3)	6.3 (5.3)	<0.001^*^	5.3 (7.4)
Sex				
Male	18 (56%)	72 (53%)	0.844	6 (50%)
Female	14 (44%)	64 (47%)		6 (50%)
Race				
White	29 (91%)	105 (79%)	0.205	8 (66.7%)
Black or African	2 (6%)	19 (14%)		2 (16.66%)
American Indian or Alaska native	1 (3%)	4 (3%)		2 (16.66%)
Asian	0 (0%)	1 (1%)		0 (0%)
Native Hawaiian or other pacific islander	0 (0%)	1 (1%)		0 (0%)
Other	0 (0%)	3 (2%)		0 (0%)
Ethnicity				
Hispanic or Latino	0 (0%)	4 (3%)	1.000	0 (0%)
Not Hispanic or Latino	31 (100%)	126 (97%)		12 (100%)
Discharge disposition				
AMA	0 (0%)	2 (2%)	<0.001^*^	0 (0%)
Expired	0 (0%)	4 (3%)		0 (0%)
Inpatient	0 (0%)	2 (1%)		1 (8.3%)
Home	26 (81%)	65 (48%)		6 (50%)
LTACH	0 (0%)	2 (2%)		0 (0%)
Nursing facility	0 (0%)	6 (4%)		1 (8.3%)
Rehabilitation	5 (16%)	52 (38%)		4 (33.3%)
Transfer	1 (3%)	3 (2%)		0 (0%)

Data is presented as mean and +/- standard deviation or n and percentage in parentheses. Statistical significance column (P-value) is based on comparison between MoCA ≥ 26 and MoCA < 26. Statistical tests conducted were Chi-Square test of independence for categorical variables, and unequal variance t-test (Welch's t-test) for continuous variables. Statistical significance threshold was set a *p* < 0.05 (^*^). MoCA, Montreal Cognitive Assessment; AMA, discharge against medical advice; LTACH, long-term acute care hospital.

**Table 2:** Stroke characteristics.

Variable	MoCA ≥26	MoCA < 26	*P*-value	Previous dementia
Lesion location				
Cerebellum	3 (10%)	8 (6%)	0.596	1 (8.3%)
Frontal	9 (28%)	51 (38%)		5 (42%)
Insular	2 (7%)	4 (3%)		0 (0%)
Medulla	0 (0%)	2 (1%)		0 (0%)
Occipital	2 (6%)	11 (8%)		0 (0%)
Parietal	0 (0%)	4 (3%)		0 (0%)
Pons	3 (9%)	8 (6%)		1 (8.3%)
Temporal	0 (0%)	3 (2%)		0 (0%)
Subcortical	10 (31%)	35 (26%)		4 (33%)
Other	1 (3%)	8 (6%)		1 (8.3%)
Not applicable	2 (6%)	2 (1%)		0 (0%)
Vessel territory				
ACA	0 (0%)	2 (1.5%)	0.842	1 (8.25%)
Anterior choroidal	0 (0%)	1 (1%)		0 (0%)
Basilar	3 (9%)	9 (6%)		1 (8.25%)
ICA	0 (0%)	3 (2%)		0 (0%)
MCA	19 (60%)	84 (61.5%)		6 (50%)
PCA	5 (16%)	19 (14%)		1 (8.25%)
PICA	0 (0%)	5 (4%)		0 (0%)
SCA	1 (3%)	3 (2%)		1 (8.25%)
PComA	1 (3%)	1 (1%)		0 (0%)
Vertebral	1 (3%)	0 (0%)		0 (0%)
Not specified	2 (6%)	9 (7%)		2 (16%)
Side				
Left	15 (47%)	55 (41%)	0.789	4 (33%)
Right	12 (37%)	59 (43%)		6 (50%)
Bilateral	3 (10%)	11 (8%)		0 (0%)
Not specified	2 (6%)	11 (8%)		2 (17%)
Stroke subtype				
Ischemic	29 (90.6%)	128 (94.1%)	0.440	11 (91.7%)
Hemorrhage	1 (3%)	6 (5%)		1 (8%)
SAH	2 (6%)	2 (2%)		0 (0%)
TPA/MT				
Yes	8 (25%)	28 (20.6%)	0.633	2 (16.7%)
No	24 (75%)	108 (79.4%)		10 (83.3%)

Data is presented as n and percentage in parentheses. Statistical significance column (P-value) is based on comparison between MoCA ≥ 26 and MoCA < 26. Statistical tests conducted were Chi-Square test of independence. Statistical significance threshold was set a *p* < 0.05.

MoCA, Montreal Cognitive Assessment; ACA, anterior cerebral artery; ICA, internal carotid artery; MCA, middle cerebral artery; PCA, posterior cerebral artery; PICA, posterior inferior cerebellar artery; SCA, superior cerebellar artery; PComA, posterior communicating artery; SAH, subarachnoid hemorrhage; TPA/MT, tissue plasminogen activator/mechanical thrombectomy.

### 3.2 Prevalence of early post-stroke cognitive deficits increases with clinical and demographic risk factors

The prevalence of cognitive impairment varied across different demographic and clinical factors. Supplementary Table S1 describes prevalence of cognitive outcomes across these factors and number of patients affected by cognitive deficits post-stroke. Older age groups showed a higher cognitive impairment prevalence, with patients aged 80 and older having the highest prevalence (91.7%), while younger patients (< 50 years) had the lowest prevalence (55.0%; Supplementary Figure S2A).

Stroke severity, as measured by the NIHSS score, also strongly correlates with EPSCI prevalence (Supplementary Figure S2B). Patients with severe strokes (NIHSS 16+) exhibited a 100% prevalence, while those with moderate strokes (NIHSS 5–15) had a prevalence of 87.0%. In contrast, mild strokes (NIHSS 0–4) had a significantly lower EPSCI prevalence of 70.4%, reinforcing that more clinically severe stroke leads to higher cognitive impairment post-stroke.

Hospital length of stay further influences EPSCI prevalence (Supplementary Figure 2C), with longer stays associated with higher PSCI prevalence. Patients who stayed 20+ days had a PSCI prevalence of 100%, while those hospitalized for 10–19 days had a 95.0% prevalence, and those staying 5–9 days experienced an 87.5% prevalence. Conversely, shorter stays (0–1 days) had the lowest EPSCI prevalence at 66.7%.

Discharge disposition significantly impacted EPSCI, with patients discharged to non-home facilities experiencing a much higher prevalence (91.9%) compared to those discharged home (70.9%; Supplementary Figure S2D). Moreover, race played a moderate role in EPSCI prevalence with non-white individuals having an increased prevalence (90.3%) compared to white individuals (78.3%).

Other categories including stroke location, vessel affected, hemisphere affected, ischemic or non-ischemic, and use of thrombolytics did not affect the prevalence of EPSCI.

### 3.3 Clinical stroke severity, length of hospitalization discharge category and race associates with early cognitive impairment post-stroke

**Figure 2:**
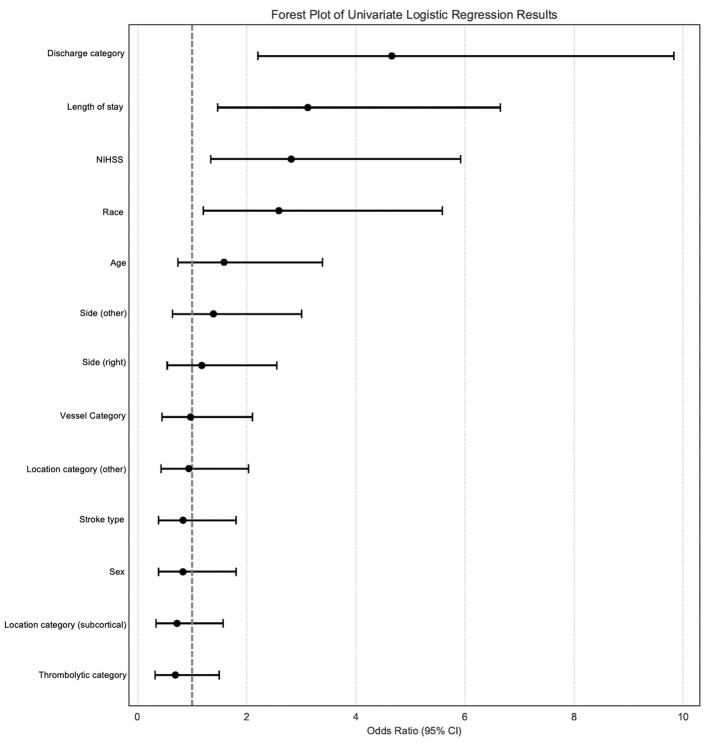
Clinical factors in univariable modeling predict EPSCI. Forest plot showing odds ratios (OR) and 95% confidence intervals (CI) for significant predictors (discharge category, length of stay, NIHSS, race) of post-stroke cognitive impairment (PSCI) from univariable logistic regression analysis.

Univariable logistic regression analysis identified multiple variables significantly associated with increased odds of EPSCI (Figure 2, Supplementary Table S3). Among continuous variables, each additional point in NIHSS score was associated with a 2.81-fold increase in the odds of EPSCI (OR = 2.814, 95% CI: 1.337–5.923). Similarly, each additional day of hospitalization increased the odds of EPSCI by approximately 3.12-fold (OR = 3.117, 95% CI: 1.462–6.646). Discharge category also played a significant role, with non-home discharge associated with 4.66-fold higher odds of EPSCI (OR = 4.658, 95% CI: 2.207–9.831). Regarding race, non-white patients had 2.59-fold increased odds of EPSCI compared to white patients (OR = 2.587, 95% CI: 1.199–5.583). Other categories did not significantly associate with increased or decreased odds of EPSCI.

### 3.4 Multivariable modeling achieves good predictive performance in detecting PSCI

**Figure 3:**
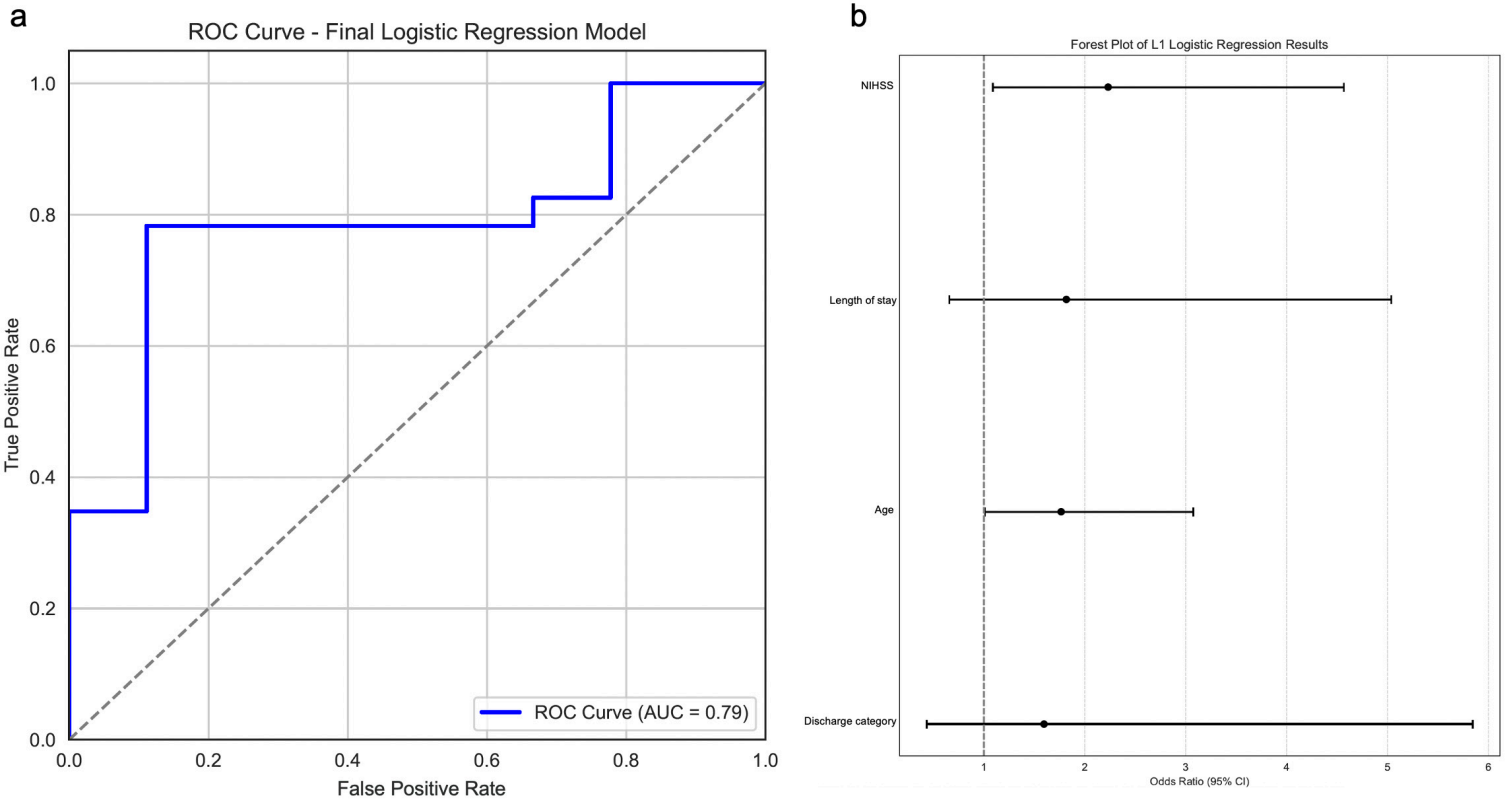
Multivariable modeling achieves good predictive performance in detecting EPSCI. **(a)** Model discrimination was assessed using the Area Under the Receiver Operating Characteristic Curve (AUC-ROC) with final model including NIHSS, length of stay, age and discharge disposition. ROC-AUC (0.7874) achieved good predictive modeling. **(b)** Forest plot showing odds ratios (OR) and 95% confidence intervals (CI) for significant predictors (NIHSS and age) of post-stroke cognitive impairment (PSCI).

The simplified multivariable logistic regression was performed to adjust for confounders and determine independent predictors of EPSCI. All non-zero predictors from the Lasso model and all statistically significant predictors from the full logistic model were included in the final model selection. The final model included NIHSS, length of stay, age, and discharge category. The model achieved a high predictive performance with a ROC-AUC of 0.7874 (Figure 3a). Stroke severity, measured by NIHSS score, remained the strongest predictor of EPSCI (OR = 2.233, 95% CI: 1.091–4.568; Figure 3b). Age was also a strong predictor (OR = 1.767, 95% CI: 1.015–3.077). Length of hospital stay, and discharge category did not remain significant predictors of PSCI in the final model (Supplementary Table S4).

### 3.5 Multivariable model with interactions did not increase the odds of PSCI

A multivariable logistic regression model incorporating interaction terms was developed to explore potential synergistic effects among predictors. Discharge disposition, NIHSS, length of stay, and age were included in this model with interaction effects between NIHSS and length of stay, NIH and age, length of stay and discharge disposition, and three-way interaction between length of stay and discharge and NIHSS. No interactions were significant (Supplementary Table S5), however, NIHSS independently remained a strong predictor in this model (OR = 5.514, 95% CI = 1.218–24.953). The full model exhibited similar predictive power (ROC-AUC = 0.7826) compared to the simpler model without interactions.

## 4 Discussion

This study found that patients with EPSCI were older, had higher NIHSS scores, experienced longer hospitalizations, and were more frequently discharged to non-home settings. Univariable analyses showed that age, NIHSS, length of stay, discharge disposition, and race were associated with EPSCI at discharge. In multivariable modeling, however, NIHSS and age remained the most robust predictors, whereas length of stay and discharge disposition did not retain statistical significance. Similarly, exploratory models incorporating interaction terms showed comparable predictive performance and did not identify significant interactions. Together, these findings support stroke severity and age as the most consistent independent predictors of early cognitive impairment in this cohort.

Our analysis demonstrated that NIHSS score was the strongest independent predictor of EPSCI, with its association remaining robust after adjustment for other variables. The influence of stroke severity on cognitive outcomes has been consistently supported in prior research. A previous study found that NIHSS scores obtained during the subacute phase significantly predicted both cognitive and functional outcomes at 3–6 months post-stroke, with higher scores correlating with worse cognitive performance (Dong et al., 2013). Similarly, another study reported that higher baseline NIHSS scores increased the risk of post-ischemic stroke disability by 28.5 times, emphasizing the central role of stroke severity in long-term cognitive prognosis (Teresa et al., 2022). Even mild strokes can have cognitive consequences, in one cohort cognitive deficits were observed in 41% of patients with minimal NIHSS scores and in all patients with NIHSS scores ≥4 (Kauranen et al., 2014). A multicenter cohort confirmed the sustained prevalence of PSCI up to 18 months post-stroke, with NIHSS at admission being a key determinant (Aam et al., 2020). Additionally, NIHSS has been identified as an independent predictor of vascular cognitive impairment (Chaudhari et al., 2014). Collectively, these findings underscore the importance of considering NIHSS not only as an acute stroke severity metric but also as a critical predictor of long-term cognitive impairment. Our data support this role, highlighting the need for early cognitive screening and proactive rehabilitation in patients with moderate-to-severe strokes.

Age emerged as the second most significant independent predictor of EPSCI in our analysis. The association between advancing age and increased risk of cognitive decline following stroke has been well-documented. A recent large-scale study from China reported that PSCI prevalence increased from 71.2% in individuals aged 19–44 to 84.2% in those aged 75 and older, illustrating the heightened vulnerability of older adults (He et al., 2023). Moreover, a recent study identified age as one of the most influential factors in models predicting PSCI at 3–6 months post-stroke (Lee et al., 2023). Consistent with these findings, our study demonstrated a stepwise increase in EPSCI prevalence across age categories, with individuals under 50 experiencing the lowest rates, and those aged 80 and above exhibiting the highest. This age-related gradient emphasizes the need for age-specific strategies in post-stroke care, including tailored cognitive monitoring and interventions in older adults who may be at elevated risk for PSCI despite similar clinical presentations. With the strong associations between age, cognitive impairment and stroke, early identification through cognitive screening post-stroke may allow for targeted cognitive therapies to mitigate PSCI prevalence long term.

To the authors' knowledge, length of hospital stay, and discharge disposition have not been previously evaluated as independent predictors of PSCI. In our analysis, both variables were significantly associated with EPSCI in univariable models; however, their significance did not persist after adjusting for covariates in multivariable modeling. Prior literature shows that both prolonged hospitalization and discharge to non-home settings often reflect greater stroke severity (Schrage et al., 2023; Kang et al., 2016). Based on this relationship, we explored potential interaction effects between stroke severity, length of stay, and discharge disposition through two-way and three-way interaction terms in our multivariable model. This approach was guided by the clinical rationale that patients with more severe strokes typically require longer hospitalizations to achieve discharge readiness and are more likely to be discharged to inpatient rehabilitation or skilled nursing facilities rather than home. Despite this plausible hypothesis, none of the interaction terms reached statistical significance. These findings suggest that while hospital course characteristics may reflect underlying stroke severity, they do not independently modify the relationship between severity and EPSCI in this dataset. Future studies with larger sample sizes are warranted to further evaluate potential interactions among these variables.

This study was limited by a relatively small sample size, which likely reduced the power to detect statistically significant interaction effects in our multivariable models. While model discrimination was assessed using ROC-AUC, we did not perform internal validation of the final model or assess calibration. Accordingly, the reported ROC-AUC should be interpreted as an in-sample estimate and not as evidence of a finalized, internally validated clinical prediction tool. Although cognitive impairment frequently manifests in the early phase after stroke (Béjot et al., 2012), some patients may experience partial cognitive recovery over time (El Husseini et al., 2023). Assessing cognition at discharge, as opposed to 3–6-month post-stroke interval, may have influenced classification of cognitive status based on MoCA scores. In addition, cognitive performance measured at discharge may be influenced by transient acute-phase factors, including delirium, fatigue, sleep disruption, and other hospitalization-related effects, rather than reflecting stable post-stroke cognitive impairment alone. Length of stay and discharge disposition are hospital-course variables measured near discharge and may partly represent downstream consequences of stroke severity rather than independent predictors of EPSCI, which limits causal interpretation of their associations. Nonetheless, identifying early markers of PSCI, more sensitive than specific, is valuable for clinical risk stratification and targeting early interventions. Notably, variables such as stroke location and involved vessel, which have been identified in prior literature as potential predictors of PSCI (Weaver et al., 2021), were not significantly associated with cognitive outcomes in our sample. This may reflect limitations in statistical power, variation in case mix, or differences in stroke subtypes. Potentially relevant factors such as educational attainment, premorbid cognitive status, and neuropsychiatric features were not accounted for in the present analysis and may have influenced the observed associations. Given the modest sample size, our multivariable approach was intentionally parsimonious; future studies with larger cohorts should evaluate these factors more directly. Similarly, the observed association with race should be interpreted cautiously, as race may act as a proxy for unmeasured social determinants of health, socioeconomic context, healthcare access, and other structural factors not captured in the present analysis, rather than reflecting biological differences. Furthermore, baseline cognitive function was not available for our cohort, which limits our ability to determine whether observed deficits were new or pre-existing, an important consideration in interpreting causality. While quantitative cognitive data were not present at baseline for the retrospective cohort, patients had no preexisting history of cognitive impairment on retrospective review.

Regarding cognitive measurement, limitations of MoCA as a cognitive assessment include use of multiple MoCA versions (30-, 25-, and 22-point formats) and the use of linear score conversion, which may introduce measurement error because the different formats assess slightly different cognitive domains and lack full psychometric equivalence in acute stroke. However, these adaptations allowed inclusion of patients with motor or visual impairments who otherwise could not complete the standard MoCA, preserving representativeness of the acute stroke population and aligning with clinical practice where adapted MoCA forms are routinely used. Lastly, a key limitation of this study is the potential for selection bias, as patients without a MoCA at discharge were excluded. During the study period, MoCA testing at discharge was not standard of care for all stroke patients at our institution, which limited the number of patients eligible for inclusion. In addition, cognitive testing can be difficult in patients with language impairment such as aphasia. A recent retrospective study from Swedish Stroke Registries found that 44% of patients did not undergo formal cognitive screening, with aphasia identified as a major factor contributing to non-assessment (Abzhandadze et al., 2021). Therefore, future research should investigate optimal cognitive screening for both fluent and non-fluent aphasias.

Our study identified length of hospital stay and discharge disposition as hospital-course variables associated with EPSCI in univariable analyses, while confirming NIHSS and age as the most robust predictors in multivariable modeling. These findings highlight the importance of considering both acute stroke severity and hospital course variables in early cognitive risk assessment. Future studies with larger, well-characterized cohorts and longitudinal follow-up should evaluate whether length of stay and discharge disposition interact with stroke severity in shaping long-term cognitive trajectories. Inclusion of baseline cognitive assessments and standardized follow-up intervals will further clarify the temporal relationship between stroke and cognitive decline.

## Data Availability

The original contributions presented in the study are included in the article/Supplementary material, further inquiries can be directed to the corresponding author.
