# Supplementary material for: NIH Stroke Scale and age predict early post-stroke cognitive impairment
Source: Front Stroke. 2026 Apr 28;5:1762758. doi: 10.3389/fstro.2026.1762758 (PMC13160796; doi:10.3389/fstro.2026.1762758)
Supplement: Supplementary file 1 [file Data_Sheet_1.DOCX]

**Supplemental Resource 1*.*** Prevalence of cognitive outcomes across clinical and demographic factors and number of patients affected by cognitive deficits post stroke.

|  |  |  | *n* | *%* |
| --- | --- | --- | --- | --- |
| *Age* | | < 50 | 20 | 55.0 |
|  | | 50-59 | 27 | 85.2 |
|  | | 60-69 | 46 | 82.6 |
|  | | 70-79 | 43 | 81.4 |
|  | | ≥80 | 24 | 91.7 |
| *NIHSS* | | 0-4 (Mild) | 71 | 70.4 |
|  | | 5-15 (Moderate) | 77 | 87.0 |
|  | | ≥ 16 (Severe) | 12 | 100.0 |
| *Length of stay* | | 0-1 days | 27 | 66.7 |
|  | | 2-4 days | 69 | 76.8 |
|  | | 5-9 days | 40 | 87.5 |
|  | | 10-19 days | 20 | 95.0 |
|  | | ≥ 20 days | 4 | 100.0 |
| *Race* | | White | 129 | 78.3 |
|  | | Other | 31 | 90.3 |
| *Ethnicity* | | Not Hispanic or Latino | 156 | 80.1 |
|  | | Hispanic or Latino | 4 | 100.0 |
| *Discharge disposition* | | Home | 86 | 70.9 |
|  | | Non-home | 74 | 91.9 |
| *Lesion location* | | Subcortical | 43 | 76.7 |
|  | | Frontal | 57 | 84.2 |
|  | | Other | 60 | 80.0 |
| *Vessel territory* | | MCA | 99 | 80.8 |
|  | | Other | 61 | 80.3 |
| *Side* | | Right | 67 | 82.1 |
|  | | Left | 67 | 77.6 |
|  | | Other | 26 | 84.6 |
| *Stroke subtype* | | Ischemic | 151 | 80.8 |
|  | | Other | 9 | 77.8 |
| *TPA/MT* | | Yes | 33 | 75.8 |
|  | | No | 127 | 81.9 |

**Note.** Abbreviations NIHSS = National Institutes of Health Stroke Scale, TPA/MT = tissue plasminogen activator/mechanical thrombectomy; values are % of patients with MoCA <26 in each category

**
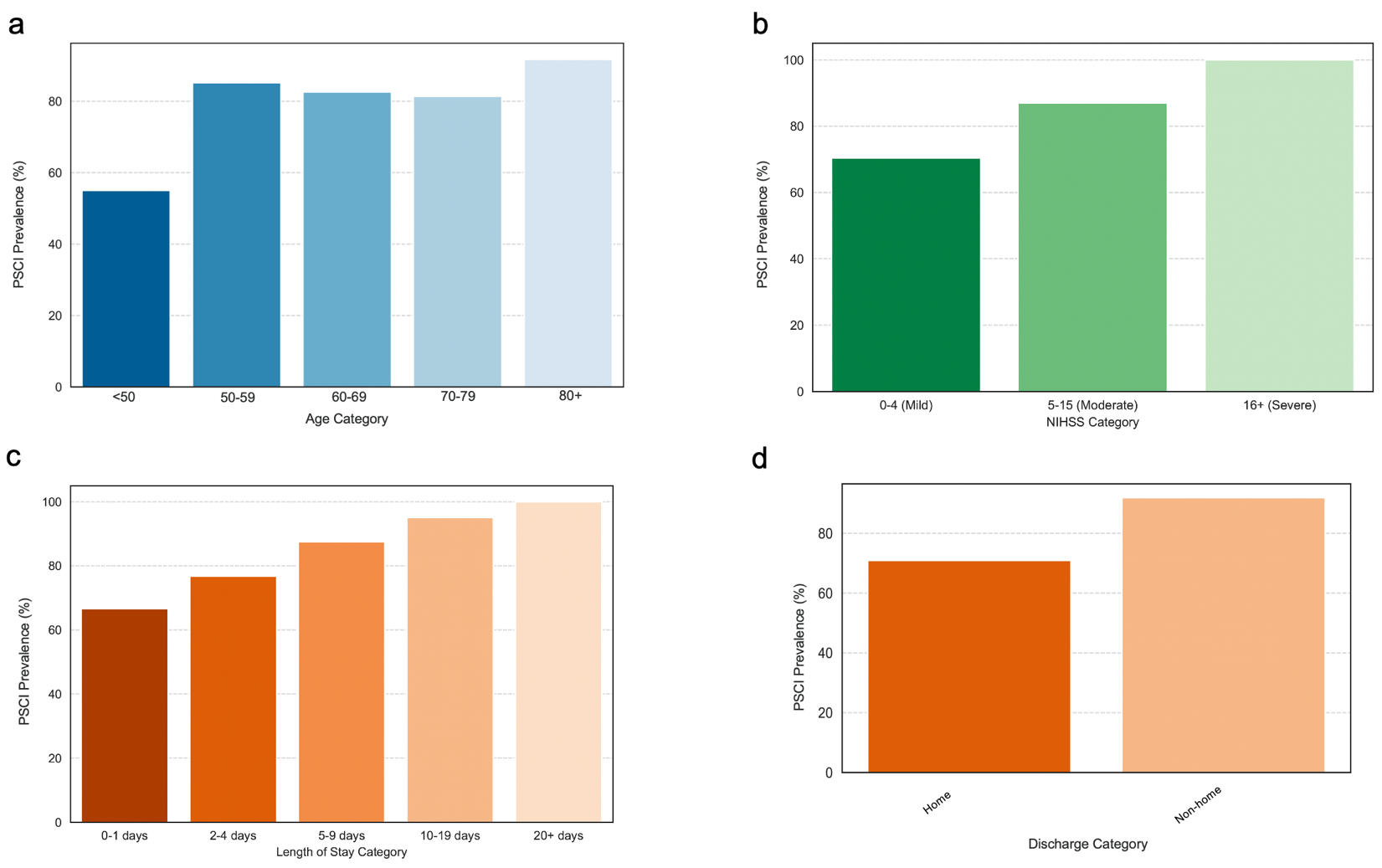
**

**Supplemental Resource 2.** Prevalence of EPSCI with clinical and demographic factors

Prevalence of early-post-stroke cognitive impairment (EPSCI) increases with older age **(a)**, higher NIHSS **(b)**, increased length of stay **(c)**, and non-home discharge disposition **(d)**.

**Supplemental Resource 3*.*** Odds ratios (OR) and 95% confidence intervals (CI) for various factors associated with clinical outcomes

|  | OR | 95% CI |
| --- | --- | --- |
| Age | 1.583 | (0.740; 3.388) |
| Sex | 0.830 | (0.383; 1.800) |
| NIHSS score | 2.814 | (1.337; 5.923)* |
| Length of stay | 3.117 | (1.462; 6.646)* |
| Race | 2.587 | (1.199; 5.583)* |
| Discharge disposition | 4.658 | (2.207; 9.831)* |
| Lesion location |  |  |
| Subcortical | 0.721 | (0.333; 1.563) |
| Other | 0.938 | (0.432; 2.035) |
| Vessel category | 0.969 | (0.447, 2.103) |
| Side |  |  |
| Right | 1.175 | (0.542; 2.548) |
| Other | 1.388 | (0.640; 3.009) |
| Stroke subtype | 0.831 | (0.383; 1.804) |
| Thrombolytic category | 0.691 | (0.319; 1.496) |

Note: * indicates p<0.05

**Supplemental Resource 4.** Multivariable logistic regression model: clinical and demographic predictors of early-post stroke cognitive impairment

| Predictor | Coefficient | SE Coefficient | Z | P | [0.025 | 0.975] |
| --- | --- | --- | --- | --- | --- | --- |
| Constant | 1.6954 | 0.435 | 3.894 | 0.000* | 0.842 | 2.549 |
| Discharge disposition | 0.4682 | 0.662 | 0.707 | 0.479 | - 0.829 | 1.766 |
| NIHSS score | 0.8031 | 0.365 | 2.198 | 0.028* | 0.087 | 1.519 |
| Length of stay | 0.5981 | 0.520 | 1.151 | 0.250 | 0.420 | 1.617 |
| Age | 0.5691 | 0.283 | 2.012 | 0.044* | 0.015 | 1.124 |

Note: Coefficients with 95% CI shown; * indicates p<0.05

**Supplemental Resource 5*.*** Multivariable logistic regression model with interaction terms: predictors of cognitive outcomes post stroke

| Predictor | Coefficient | SE coefficient | Z | P | [0.025 | 0.975] |
| --- | --- | --- | --- | --- | --- | --- |
| Constant | 2.3857 | 0.830 | 2.875 | 0.004* | 0.759 | 4.012 |
| Discharge disposition | 0.2164 | 0.836 | 0.257 | 0.796 | -1.423 | 1.856 |
| NIHSS score | 1.7072 | 0.770 | 2.216 | 0.027* | 0.197 | 3.217 |
| Length of stay | 2.2713 | 1.467 | 1.549 | 0.121 | -0.603 | 5.146 |
| Age | 0.6316 | 0.335 | 1.887 | 0.059 | -0.024 | 1.287 |
| NIHSS x age | 0.0813 | 0.366 | 0.222 | 0.824 | -0.635 | 0.798 |
| NIHSS x length of stay | 2.5340 | 1.365 | 1.857 | 0.063 | -0.141 | 5.209 |
| Discharge disposition x length of stay | -0.0766 | 1.655 | -0.046 | 0.963 | -3.320 | 3.167 |
| NIHSS x discharge disposition x length of stay | -1.0977 | 2.023 | -0.543 | 0.587 | -5.062 | 2.867 |
|  |  |  |  |  |  |  |

Note: Interaction terms as specified; * indicates p<0.05
